# Metamorphic development of the olfactory system in the red flour beetle (*Tribolium castaneum*, Herbst)

**DOI:** 10.1186/s12915-021-01055-8

**Published:** 2021-07-30

**Authors:** Björn Trebels, Stefan Dippel, Brigitte Goetz, Maria Graebner, Carolin Hofmann, Florian Hofmann, Freya-Rebecca Schmid, Mara Uhl, Minh-Phung Vuong, Vanessa Weber, Joachim Schachtner

**Affiliations:** 1 Department of Biology, Animal Physiology, Philipps-University Marburg, Karl-von-Frisch-Str. 8, 35032 Marburg, Germany; 2 Clausthal University of Technology, Adolph-Roemer-Str. 2a, 38678 Clausthal-Zellerfeld, Germany

**Keywords:** *Tribolium castaneum*, Olfaction, Insect, Metamorphic development, Antennae, Antennal lobe, Gnathal olfactory center, Neuroanatomy

## Abstract

**Background:**

Insects depend on their olfactory sense as a vital system. Olfactory cues are processed by a rather complex system and translated into various types of behavior. In holometabolous insects like the red flour beetle *Tribolium castaneum*, the nervous system typically undergoes considerable remodeling during metamorphosis. This process includes the integration of new neurons, as well as remodeling and elimination of larval neurons.

**Results:**

We find that the sensory neurons of the larval antennae are reused in the adult antennae. Further, the larval antennal lobe gets transformed into its adult version. The beetle’s larval antennal lobe is already glomerularly structured, but its glomeruli dissolve in the last larval stage. However, the axons of the olfactory sensory neurons remain within the antennal lobe volume. The glomeruli of the adult antennal lobe then form from mid-metamorphosis independently of the presence of a functional OR/Orco complex but mature dependent on the latter during a postmetamorphic phase.

**Conclusions:**

We provide insights into the metamorphic development of the red flour beetle’s olfactory system and compared it to data on *Drosophila melanogaster*, *Manduca sexta*, *and Apis mellifera*. The comparison revealed that some aspects, such as the formation of the antennal lobe’s adult glomeruli at mid-metamorphosis, are common, while others like the development of sensory appendages or the role of Orco seemingly differ.

**Supplementary Information:**

The online version contains supplementary material available at 10.1186/s12915-021-01055-8.

## Background

In insects, olfactory perception usually starts at the chemosensory sensilla of the antennae and palps. The sensilla house the chemosensory neurons (CSNs). CSNs divide into olfactory sensory neurons (OSNs) and gustatory sensory neurons (GSNs). The OSNs present the olfactory receptors, either odorant receptors (ORs) or ionotropic glutamate-like receptors (IRs), in their membranes [1–5]. Notably, most sensory information received by the OSNs of insects stems from a functional heteromer of specific odorant receptors (ORs) and the odorant receptor co-receptor (Orco) [6]. Thus, elimination of Orco typically leads to a loss of most olfactory transduction [7–12].

The OSNs then relay the olfactory information via their axons to the respective primary processing centers. For the antennal OSNs, these centers are the antennal lobes, while those of the palpal OSNs differ among species. In hemimetabolous insects, the signal from palpal OSNs gets processed in the glomerular lobes (LGs) [13]. The current picture of olfaction in holometabolous insects states that the palpal signals are also processed in the ALs [14–18]. However, at least in the red flour beetle, the palpal OSNs do not project into the ALs but into the paired LGs and the unpaired gnathal olfactory center (GOC), which is a glomerularly organized neuropil within the gnathal ganglion [5].

In holometabolous insects, the lifestyle of imagines and larvae typically differs. Consequently, during metamorphosis, the olfactory system’s morphology is remodeled to reflect the new challenges. Already the larvae of holometabolous insects possess olfactory appendages, while the complexity of the primary processing centers differs among species. The larvae of *Tribolium castaneum* possess elaborate antennae with three distinguishable segments (scape, pedicel, flagellum) [19, 20], which is also described for the red flour beetle’s close relative *Tenebrio molitor* [21] and some other beetles [22–24]. The larval antennae of the hawkmoth *Manduca sexta* are similar in structure [25, 26], whereas the larvae of flies only possess functional equivalent dorsal organs [27, 28].

The adult antennae of the vinegar fly *Drosophila melanogaster* [29, 30] and the hawkmoth *Manduca sexta* [31] are built from imaginal disks during the pupal stage, whereas the antennae of the hemimetabolous American cockroach *Periplaneta americana* grow gradually with every molt [32, 33]. Previous studies on appendage development in holometabolous insects focused on species with imaginal discs or cells. However, it is discussed that reusing larval appendages to build their adult equivalents is the more ancestral state [34]. This mechanism is found during the development of the adult legs of the red flour beetle [35], of which the antennae are serial homologs [36]. We used transgenic lines labeling CSNs and OSNs, in combination with cell birth detection, to visualize and follow the sensory neurons of the antennae throughout pupal formation and metamorphosis to investigate the origin of the beetle’s adult antennae.

Previous studies showed that the organization of the larval antennal lobes (ALs) differs between holometabolous insect species. In the lepidopteran *M. sexta* and the hymenopteran *Apis mellifera,* the larval ALs are not glomerularly organized [37, 38], whereas the larval ALs of *the mealworm beetle Tenebrio molitor* are glomerularly organized [39]. Further, the larval ALs of *D. melanogaster* possess glomeruli but with a lower count and one-to-one wiring between receptor neurons and glomeruli [40]. Besides, in the hemimetabolous American cockroach *Periplaneta americana,* the ALs show similar numbers of glomeruli in nymphs (larvae) and adults [41].

So far, studies indicate that OSNs are required for the proper formation of the adult AL glomeruli. In *M. sexta,* de-antennation leads to non-glomerular ALs [42]. Further, in the clonal raider ant *Ooceraea biroi,* de-antennation leads to a heavily reduced glomeruli number [43]. In the ant, the same result was achieved in Orco knock-out experiments [44, 45]. The authors suggest that the effect is more likely due to the loss of the majority of OSNs caused by the knock-out. Knock-out studies on the metamorphic development of the ALs of *D. melanogaster* indicate that activity of the OR/Orco complex is not necessary for the formation of AL glomeruli [7, 46, 47], which is also reported for the malaria mosquito *Anopheles gambiae* [48].

In our study, we used (immuno)-histochemistry to visualize the formation of the adult glomeruli of the ALs and the GOC of the red flour beetle. We took advantage of the strong dsRNA injection-induced systemic RNA interference [49–51] to effectively knockdown Orco just before the pupal formation to study the role of Orco on the formation of the AL glomeruli in *T. castaneum*.

Within the ALs, the olfactory information perceived by the OSNs is processed by a network of glomeruli connecting local interneurons (LNs). Olfactory representations, shaped by the LNs, are mainly due to the inhibitory transmitter gamma amino-butyric acid (GABA), the excitatory transmitter acetylcholine [52–65], and numerous neuropeptides [13, 66–68]. As evident from *D. melanogaster* [60] and many other insects [13, 69], the vast majority of LNs use the inhibitory transmitter GABA, therefore providing a good estimate for LN numbers.

Insight into the LN development in beetles is provided for *T. molitor*, a close relative of *T. castaneum*. In *T. molitor,* GABA expressing LNs (somata in the cluster “CL7”) are present in larvae, remain present with similar numbers with the onset of metamorphosis, and eventually rise in numbers throughout metamorphosis [39]. To study the development of the AL LNs in the red flour beetle, we labeled GABAergic neurons by immunohistochemistry against glutamic acid decarboxylase (GAD), which catalyzes the decarboxylation of glutamate to GABA. We determined the number of GAD immunoreactive cells in a lateral cluster comparable to the cluster “CL7” in *T. molitor* and used reliable neurogenesis detection with EdU [10] to determine their origins.

## Results

### Antennae, sensory neurons, and antennal lobe glomeruli

**Fig. 1:**
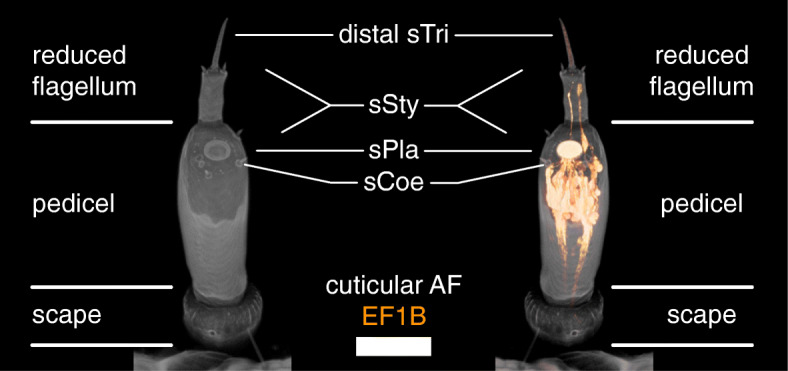
Structure of the larval antenna. Volume rendering of a confocal image stack. Depicted in gray the cuticular autofluorescence and orange the reporter signal in the EF-1-B-DsRed line representing CSNs. sTri – trichoid sensilla, sSty – styloconic sensilla, sPla – placoid sensilla, sCoe – coeloconic sensilla. Scale bar 50 μm

**Fig. 2:**
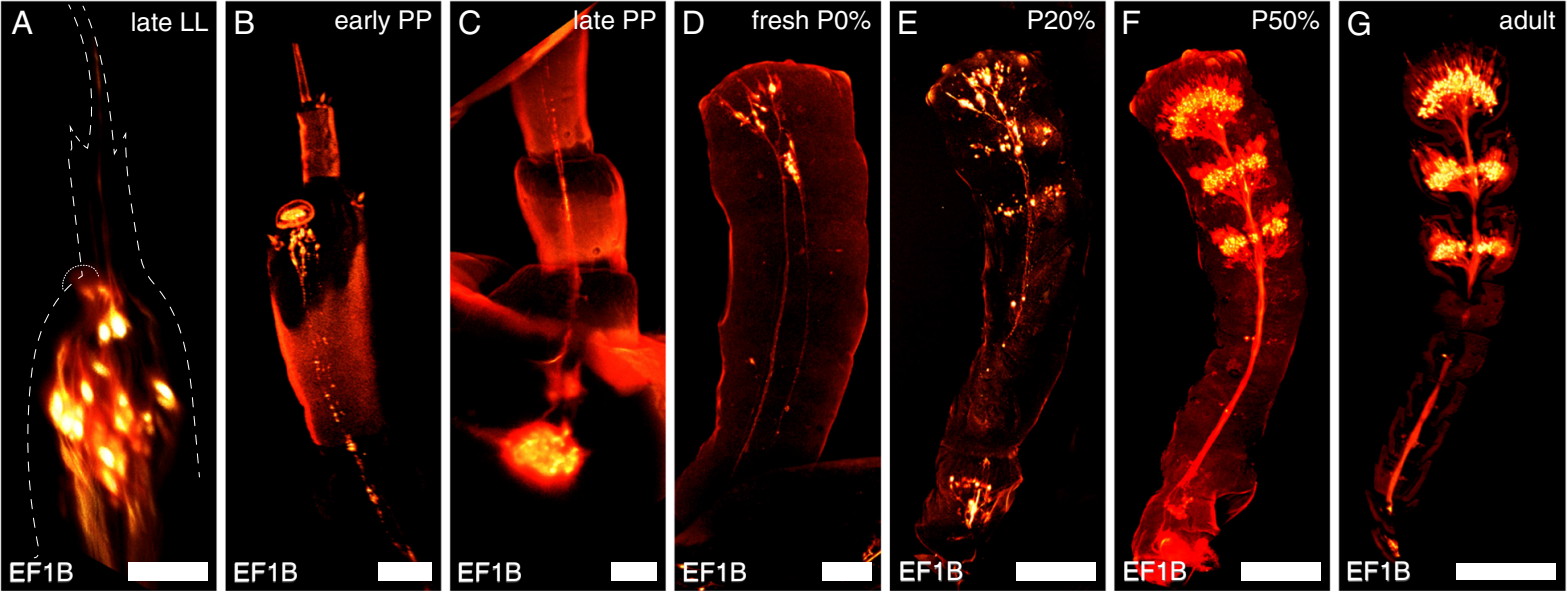
Development of the antennal chemosensory neurons (CSNs). Volume rendering of the reporter signal in the antenna at different developmental stages in the EF-1-B-DsRed line. **A** The dashed line depicts the outline of the antenna based on the autofluorescence of the cuticula. In the last larval instar (LL), CSNs innervate the placoid and the distal trichoid sensillum. **B**, **C** The neurons retract from the larval antenna during the prepupa (PP), and the somas relocate into the head capsule. **D** With the onset of metamorphosis, CSNs are found at the distal end of the adult antennae. **E**–**G** Their number rises until stage P50% when their gross distribution already resembles that of the adult antenna. Scale bars **A**–**C** 25 μm; **D**–**G** 100 μm

**Fig. 3:**
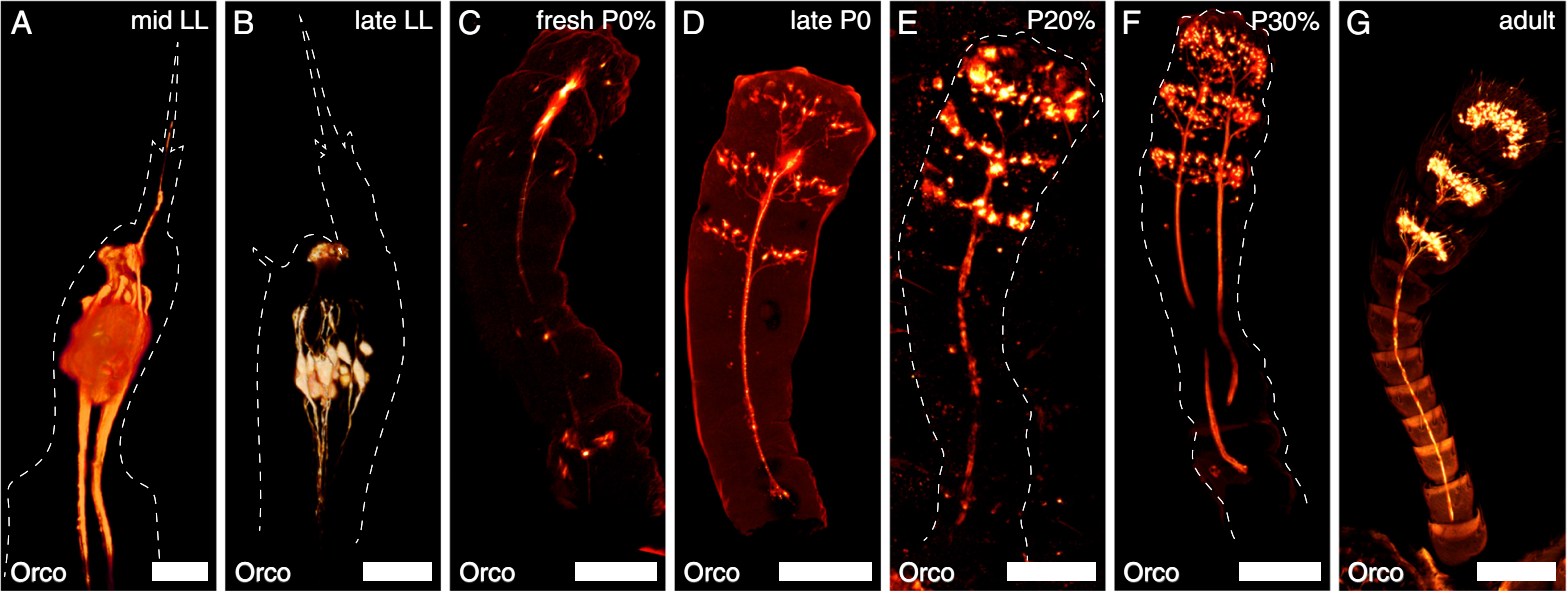
Development of the antennal olfactory sensory neurons (OSNs). Volume renderings from confocal image stacks of the reporter signal in the antenna at different developmental stages within the Orco-GAL4xUAS-DsRed line. The dashed lines depict the outline of the antenna based on the autofluorescence of the cuticula. **A**, **B** In the last larval instar (LL), OSNs innervate the placoid and the distal trichoid sensillum. **C** The OSNs retract from the larval antenna during the prepupa (PP) and are with the onset of metamorphosis found at the distal end of the adult antenna. **E**–**G** Their number rises until P50% when their distribution already resembles the adult antenna. Scale bars **A**, **B** 25 μm; **C**–**G** 100 μm. Two time-lapse videos of approx. the first 30 h of OSN-development are available as Additional file 3: Video S1 and Additional file 4: Video S2

The *T. castaneum* larvae possess a pair of three-parted antennae, each consisting of scape, pedicel, and a reduced flagellum (Fig. 1) [19, 20]. The distal trichoid sensillum (sTri) and the placoid sensillum or plate organ (sPla) of the larval antenna are both labeled in the CSN-labeling EF-1-B-DsRed line (Fig. 2), as well as in the OSN-labeling partial Orco-Gal4xUAS-DsRed line (Fig. 3A).

**Fig. 4:**
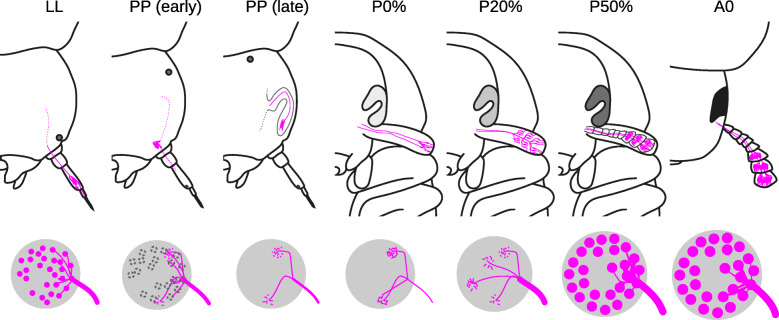
Metamorphic development of the *T. castaneum* antennae and antennal lobes. The upper row visualizes the location of the OSNs (magenta) within the antennae and head capsule, while the lower row displays the OSN axons and their arborizations within the ALs, as well as the state of AL glomerulization. LL - last larval instar, PP - prepupa

Late during the last larval instar and within the first few hours of the prepupal stage, the CSNs retract their dendrites. Further, their somata relocate into the head capsule, where they are detectable in later prepupal stages (Figs. 2B, C; 3B; 4). At this time, the gross morphology of the adult antenna is already found beneath the larval cuticle (Fig. 4; Additional file 1: Figure S1 A, B)—with the labeled CSNs (Additional file 1: Figure S1 A’, C) and OSNs (Additional file 1: Figure S1 D) located in its tip (Fig. 4, Additional file 1: Figure S1 A’). As the partial Orco-Gal4xUAS-DsRed line labels fewer neurons, which results in a much weaker signal, and as imaging requires scanning through two cuticles (larval and pupal), we were not able to acquire confocal stacks of the OSNs in prepupae suitable for volume rendering as provided for the CSNs.

**Fig. 5:**
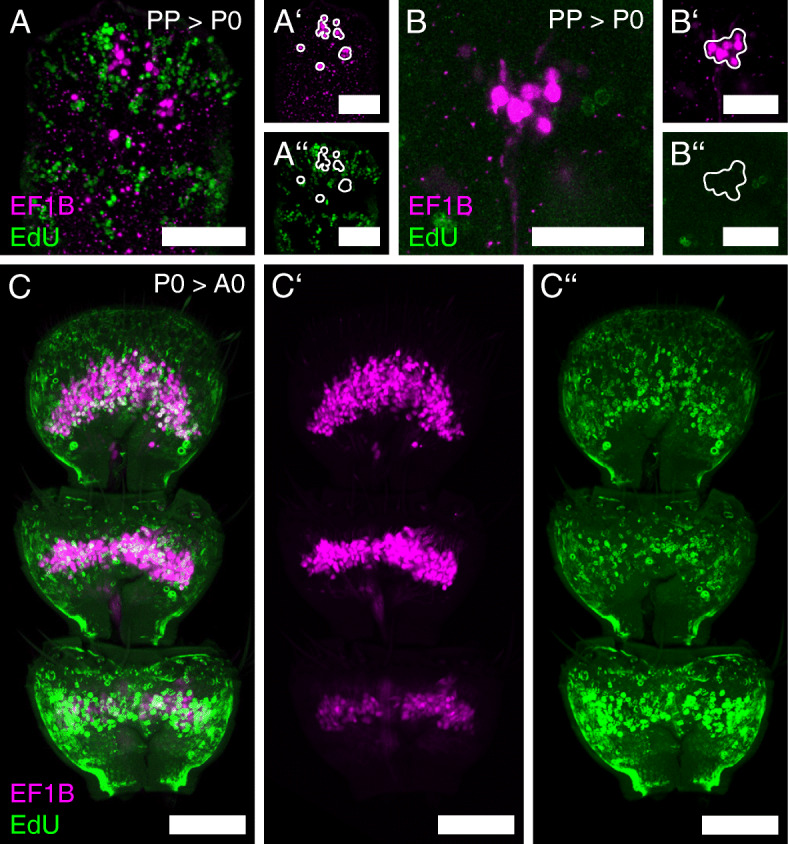
Genesis of the antennal CSNs. Antennal maximum projections of the reporter signal in the EF-1-B-DsRed line (magenta), as well as EdU-labeled cells (green). **A**-**A’’**, **B**-**B’’** After EdU injection in the prepupa (PP), none of the CSNs in the antenna at P0% is labeled with EdU. **C**-**C’’** After EdU injection at P0%, a large amount of CSNs in the adult antenna at A0 is labeled. Scale bars **A**-**A’’**, **C**-**C’’** 50 μm, **B-B’’** 25 μm


Simultaneously, OSN axons are found within the antennal lobe (Fig. 4; Additional file 2: Figure S2). During the last hours of pupal stage P0%, the gross distribution of OSNs in the last three segments of the flagellum becomes readily visible (Figs. 2d and 3D, Additional file 3: Video S1 and Additional file 4: Video S2). During the following stages, the OSN number rises (Figs. 2E, F and 3E, F; Additional file 3: Video S1 and Additional file 4: Video S2) and mostly resembles the adult distribution (Figs. 2G and 3G) at about P50% (Fig. 2F). EdU injections into the prepupa with subsequent dissection at P0% revealed that the CSNs found in the antennae of fresh pupae are born before pupation (Fig. 5A, B), while EdU injections at P0% and detection at A0 revealed that the majority of adult CSNs are born during metamorphosis (Fig. 5C).

**Additional file 3: Video S1.** Timelapse of OSN development (whole head capsule). Visualized by the fluorescent reporter in the Orco-Gal4 x UAS-DsRed line covering approximately the first 30 hours of metamorphosis.


**Additional file 4: Video S2.** Timelapse of antennal OSN development (single antenna). Visualized by the fluorescent reporter in the Orco-Gal4 x UAS-DsRed line covering approximately the first 30 hours of metamorphosis.

**Fig. 6:**
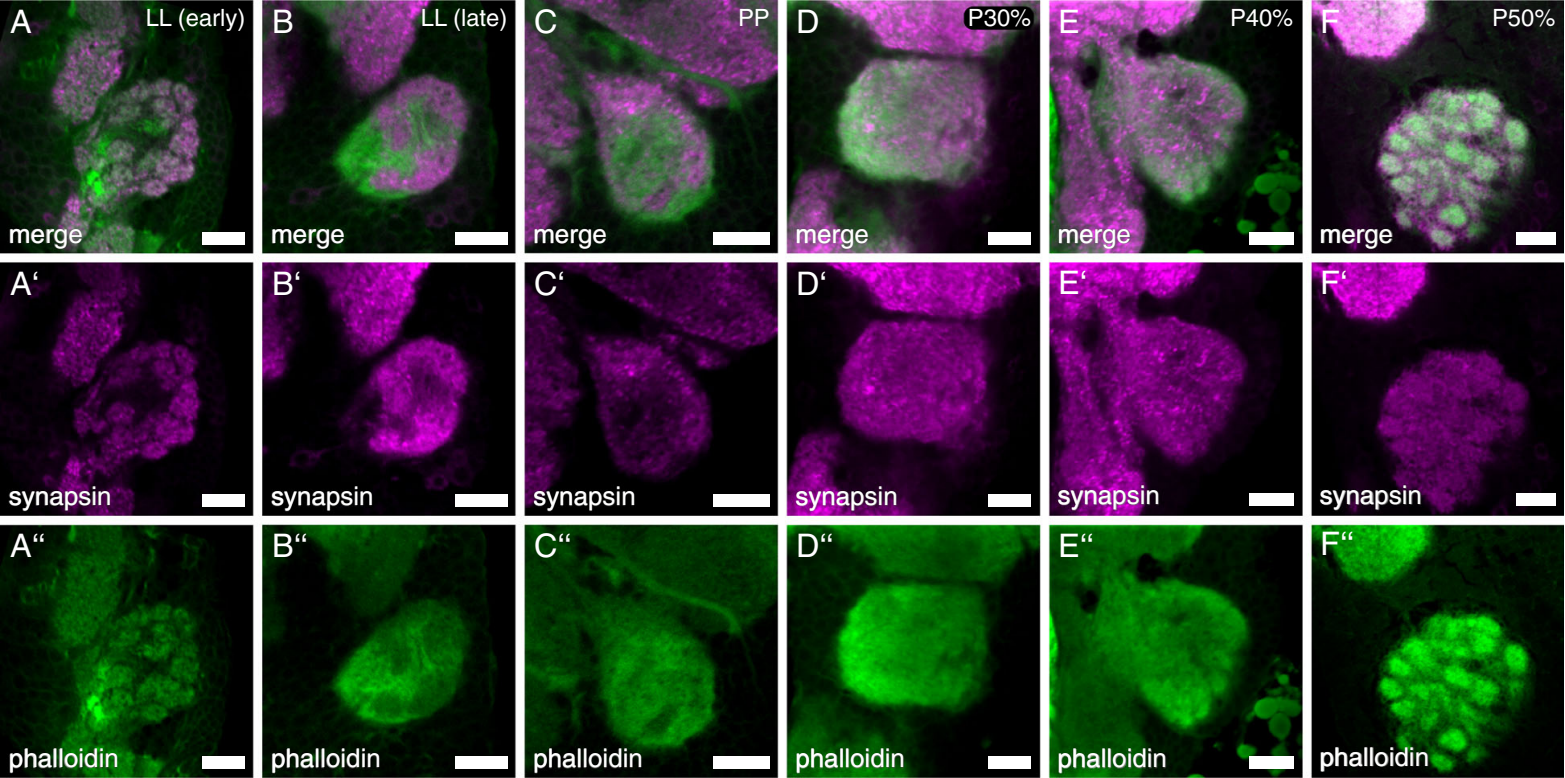
Development of the adult antennal lobe glomeruli. Representative optical slices of the antennal lobes of the red flour beetle *T. castaneum* at different developmental stages (LL - last larval instar, PP - prepupa, P - pupal stages), visualized by synapsin (magenta) and phalloidin (green) staining. Scale bars 10 μm

In the early phase of the last instar larvae, the about 45 AL glomeruli (mean = 44.75, SD = 3.42; N_ALs_ = 12) are defined in the f-actin and synapsin labeling (Fig. 6A-A’’). The glomerular organization vanishes during the late phase of last instar larvae in the f-actin and synapsin labeling (Fig. 6B-B’’). In the prepupa up to pupal stage P30%, a glomerular organization within the antennal lobe is absent (Fig. 6C-C’’, D-D’’). At pupal stage P40% (Fig. 6E-E’’), glomerulization becomes visible in the f-actin staining, with incipient glomerulization visible in the synapsin staining. At mid-metamorphosis (P50%; Fig. 6F-F’’), glomerulization is obvious in the f-actin staining. In the synapsin staining, weak yet distinct glomerulization is visible. At this stage, the antennal lobes consist of about 70 (mean = 68.44, SD = 1.89; N_ALs_ = 9) glomeruli, which is also the number found in freshly eclosed (A0) beetles [70].

### Role of Orco during the formation of the antennal lobe glomerular map

**Fig. 7:**
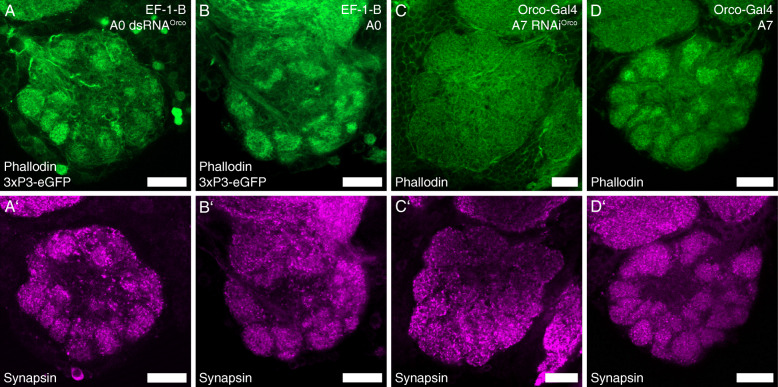
Antennal lobe (AL) glomeruli after larval *RNAi*^*Orco*^*. R*epresentative single optical slices of the AL of freshly eclosed (A0) beetles of the EF-1-B line (**A**, **A’**, **B**, **B’**) and 7-day-old beetles (A7) of the Orco-GAL4 line (**C**, **C’**, **D**, **D’**) after *dsRNA*^*Orco*^ injection in late larvae with comparison to wildtype beetles visualized by phalloidin (**A**–**D**; green) and synapsin (**A’**–**D’**; magenta). **A**, **B** In freshly eclosed beetles, the AL glomeruli are clearly visible in the phalloidin and synapsin staining under both conditions. **c**, **d** At A7 glomeruli are clearly visible in both stainings in untreated beetles, while glomeruli in knockdown beetles are hardly visible in the phalloidin staining, and only vague glomeruli are visible in the synapsin staining. Scale bars 20 μm

**Fig. 8:**
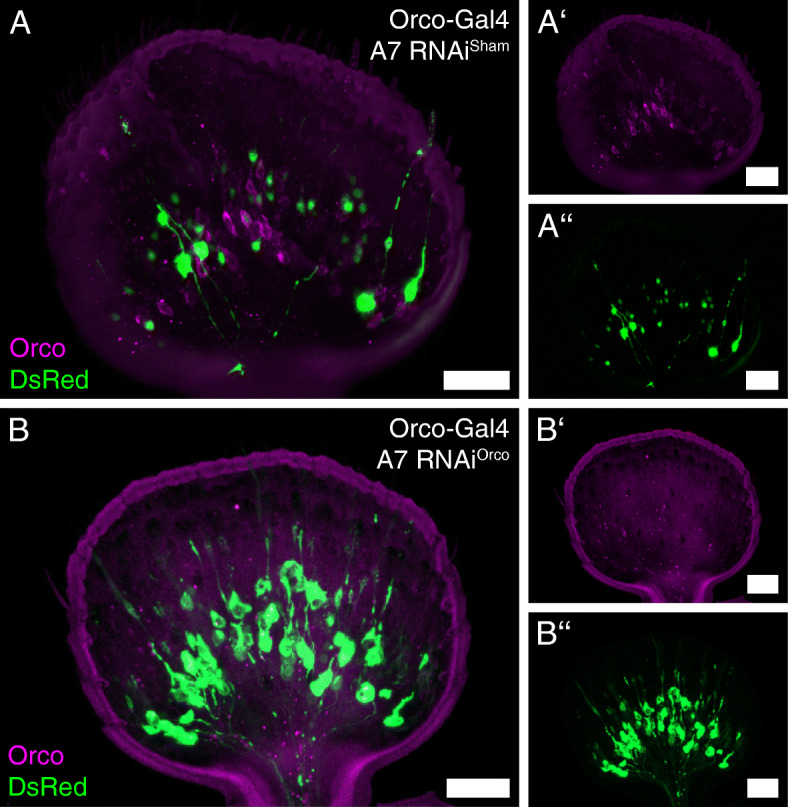
Olfactory sensory neurons and Orco immunoreactivity in the antennae after larval *RNAi* induction. Representative maximum projections of 50 μm cryo-section of antennae of seven days old beetles (A7) in the Orco-GAL4 line after *dsRNA*^*Sham*^ (**A**-**A’’**) and *dsRNA*^*Orco*^ (**B**-**B’’**) injection in late larvae. The reporter signal of the transgenic line is depicted in green, while the Orco immunostaining is depicted in magenta. Scale bars 20 μm

We find Orco-expression in pupae already before glomerulization of the adult ALs starts (Fig. 3, Additional file 5: Figure S3). In experiments using dsRNA-injection-induced systemic RNA interference (RNAi) against Orco and thereby effectively blocking OR/Orco driven olfactory transduction [10], we find that a knockdown of Orco induced in late larvae seemingly does not block the formation of the olfactory glomeruli. The AL glomeruli are still easily distinguishable in freshly eclosed (A0) knockdown beetles (*n* = 8; Fig. 7A) as they are in wildtype beetles (*n* = 7; Fig. 7B; compare also [70]). Further, the same experiments showed that in 7-day-old (A7) beetles, glomerulization is heavily reduced in knockdown beetles (*n* = 7; Fig. 7C), while they are clearly visible in the wildtype (*n* = 7; Fig. 7D; compare also [5, 67]). However, even in 7-day-old knockdown beetles, the OSNs still locate in the antennae, with their dendrites within the olfactory sensilla (Fig. 8B).

### Local neurons of the antennal lobe (AL)

**Fig. 9:**
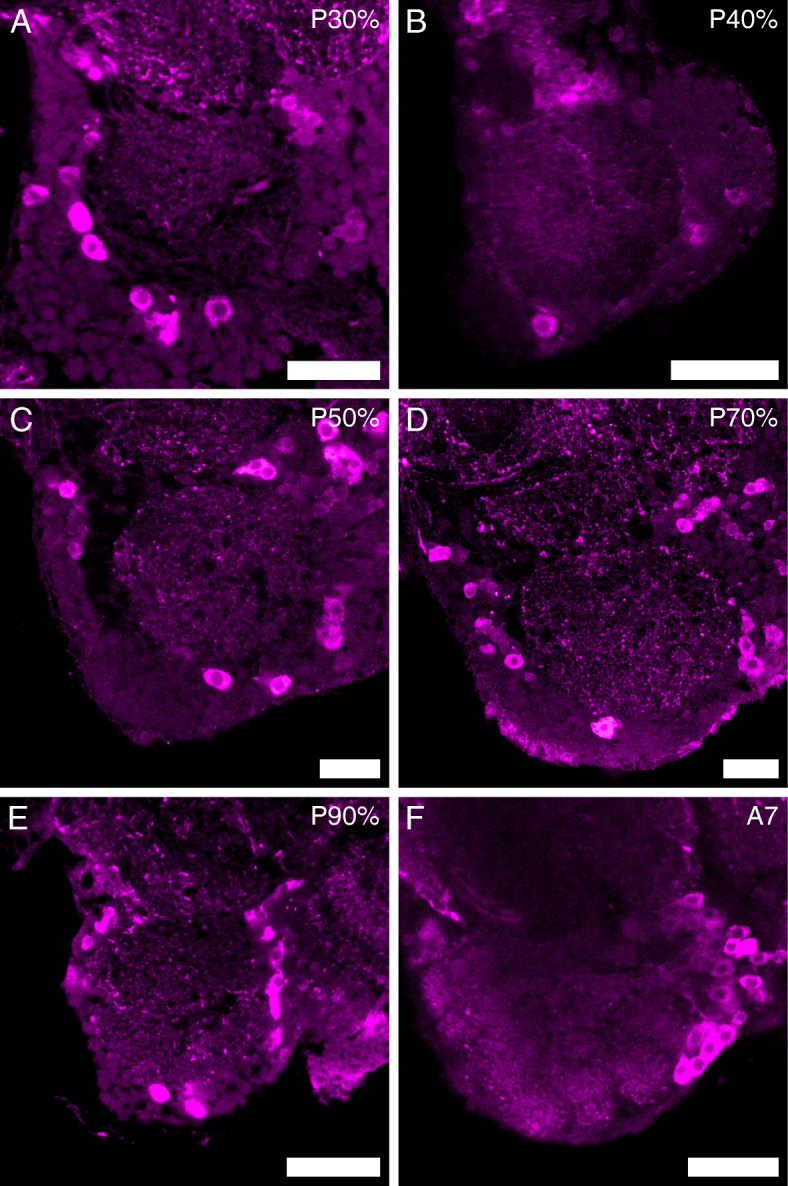
GAD immunostaining in the developing AL. Representative single optical slices of the antennal lobes stained against GAD (pupae: GADr; adults: GADs) at different time points during metamorphosis (P30%, P50%, P70%, P90%) and adult stage A7. Scale bars 20 μm

In *D. melanogaster* [60] and many other insects [13, 69], the vast majority of the AL LNs use the inhibitory transmitter GABA, which is synthesized by GAD. We used immunohistochemistry against GAD (pupae: GADr; adults: GADs) to follow the development of the AL LNs. From at least P30%, the antennal lobes are innervated by GAD immunoreactive fibers, while a distinct glomerular pattern in the GAD immunostaining is first visible in adult stage A7 (Fig. 9). The number of GAD immunoreactive somata rises during metamorphosis. At pupal stages P30% and P40%, about 65 (P30%: mean = 64.00, SD = 5.72; N_ALs_ = 7; P40%: mean = 65.75, SD = 1.71, N_ALs_ = 4) immunoreactive cells locate in the lateral cluster of each antennal lobe. At pupal stage P50%, the clusters consist of about 70 cells each but display a high variation (mean = 70.40, SD = 49.30, N_ALs_ = 5). At P70% about 130 cells (mean = 130.83, SD = 4.96, N_ALs_ = 6) and at P90% about 155 cells (mean = 154.5, SD = 11.79, N_ALs_ = 4) are found in each lateral cluster. The number then rises to about 165 cells in 7-day-old adult beetles (A7; mean = 164.3, SD = 23.46, N_ALs_ = 10).

Injection of EdU at P0% with dissection at A0 and EdU injection at different metamorphic stages (P20%, P50%, P70%, P80%) with subsequent dissection after 24 h did not result in labeled neurons within the AL, while in the same specimen labeled cells and thus presumably newborn neurons are found in the mushroom bodies and optic lobes (data not shown).

### Glomeruli of the gnathal olfactory center (GOC)

**Fig. 10:**
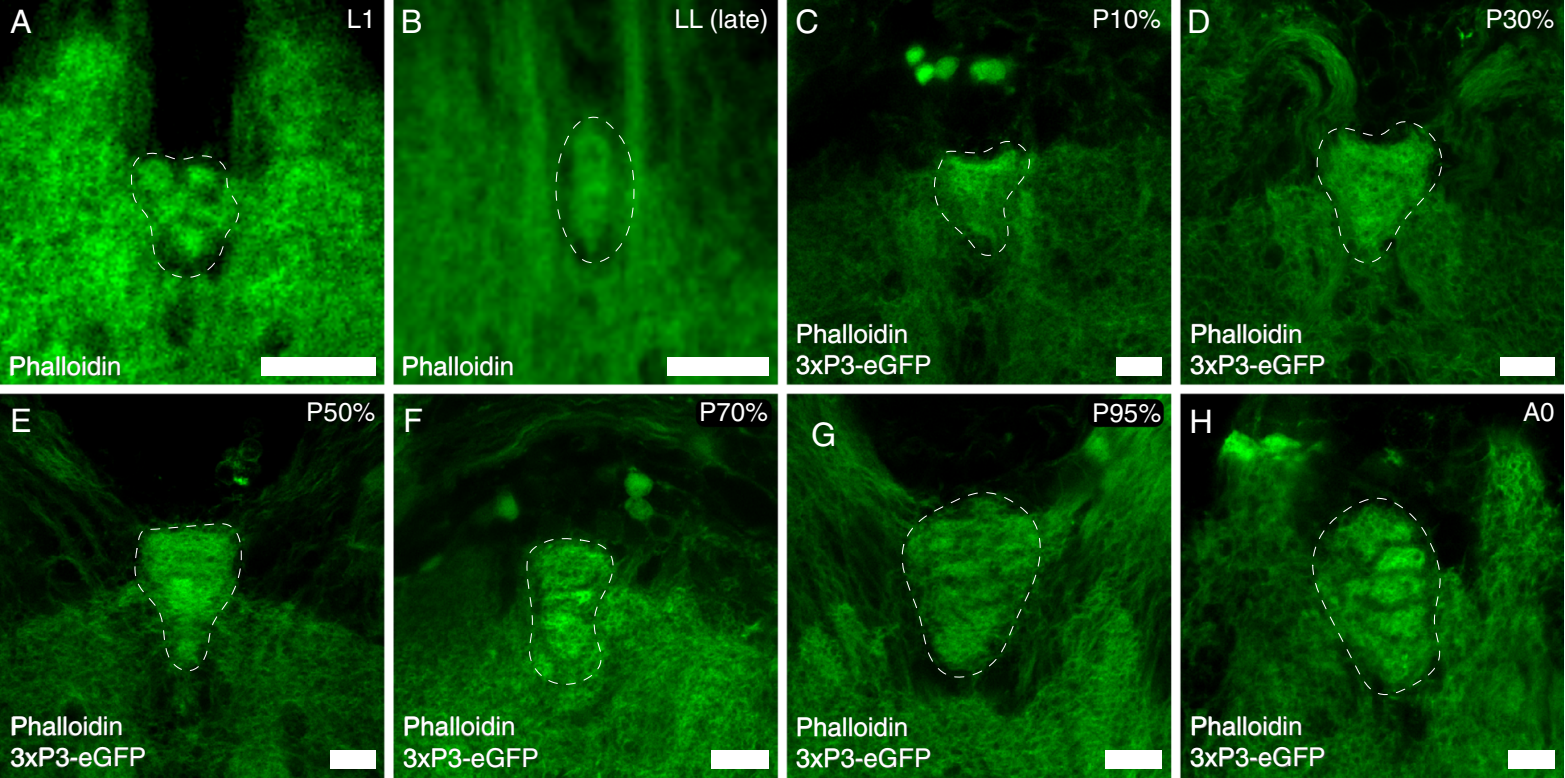
Glomeruli formation within the gnathal olfactory center. Representative optical slices of the GOC at different developmental stages (L1 - first larval instar, LL - last larval instar, P - pupal stages, A0 - freshly eclosed), visualized by **A**, **B** phalloidin in the SB line and **C**–**H** phalloidin plus 3xP3-eGFP in the EF-1-B-DsRed line. Scale bars 10 μm

In the red flour beetle, palpal olfactory input is not processed within the AL as in *D. melanogaster* but in the LGs and the GOC [5]. The GOC is already present as glomerularly organized neuropil in the first larval stage (L1) (Fig. 10A). The glomeruli vanish during the last larval stage (Fig. 10B) and are no longer distinguishable in the early pupae (P10%; Fig. 10C). At pupal stage P30%, a non-glomerular GOC is clearly distinguishable in the phalloidin staining (Fig. 10D). At about mid-metamorphosis (P50%), incipient glomerulization becomes visible in the phalloidin staining (Fig. 10E). About 30 h later, at stage P70% (Fig. 10F), the glomerulization becomes more clearly visible in the f-actin staining (Fig. 10F). Glomerulization is clearly visible another 35 h later at P95% (Fig. 10G).

### *Apis mellifera* AL LNs

In adult honeybees, all AST-A immunoreactive neurons are co-labeled with an anti-GABA antiserum. They are a subpopulation of the inhibitory GABA local neuron network in the ALs [71]. We used immunohistochemical labeling of AST-A expressing neurons to follow the development of the AL LN subpopulation.

In pupal stage P1 (P10%), first, stained fibers are visible in the AL (Additional file 7: Figure S5 A). First somata become visible at stage P2 (P20%; Additional file 7: Figure S5 B, C), and their number rises after glomeruli formation. Incipient glomeruli formation becomes first visible in the AST-A staining at P4 (P40%; Additional file 7: Figure S5 D). At P50%, glomerulization is obvious (Additional file 7: Figure S5 E). Innervation of the olfactory glomeruli then increases throughout metamorphosis (Additional file 7: Figure S5 F, G) and eventually reaches the adult pattern (Additional file 7: Figure S5 H).

## Discussion

**Fig. 11:**
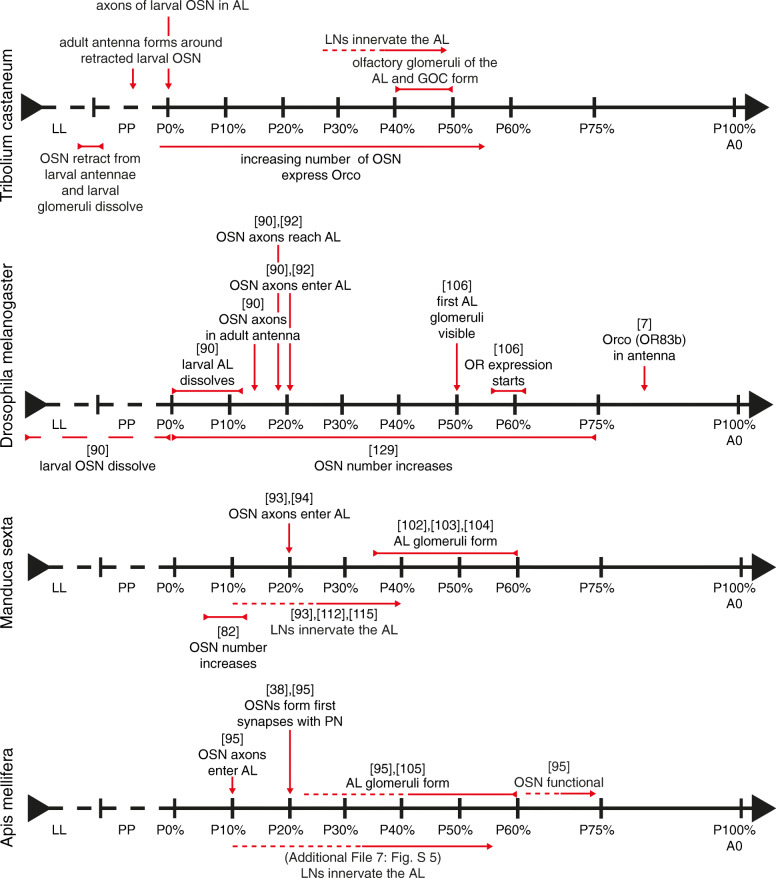
Comparison of olfactory system metamorphosis in three insect models

The anatomy of the adult olfactory system of the red flour beetle *T. castaneum* is well described [5]. To date, information about the metamorphic development and the origin of the structures of the olfactory system in *T. castaneum* is not and in beetles generally rarely available [39, 72]. In the current developmental study, we asked at which stage of metamorphosis the structures of the olfactory system form and aimed to reveal the origins of OSNs and LNs. To accomplish this, we used a combination of immunohistochemical staining, reporter expression in the CSN-labeling EF-1-B-DsRed [73], and the OSN-labeling partial Orco-GAL4xUAS-DsRed [5] line, as well as neurogenesis detection with EdU [10] and systemic RNAi against *Orco*. An overview of the developmental events including an interspecies comparison is given in Fig. 11.

### Development of the antennae and their sensory neurons

Starting at the periphery, we find a segmented larval antenna consisting of scape, pedicel, and a reduced flagellum (Fig. 1), as already described earlier [19, 20]. Analysis of the reporter signal in the transgenic lines showed that the distal large trichoid sensillum functions in chemosensation. This result fits previous studies that supposed a function in contact chemoreception [19]. Trichoid sensilla, in general, might also act in mechanoreception and/or airborne chemoreception [74, 75]. As the distal large trichoid sensillum was not only labeled in the CSN-labeling EF-1-B DsRed line but also the OSN-labeling Orco-Gal4xUAS-DsRed line, we further suggest an olfactory function (Fig. 3A). This corresponds to the fact that also the adult antennae possess olfactory trichoid sensilla [5]. However, the main olfactory function of the larval antennae is provided via the placoid sensillum (Fig. 3A). It is considered a fusion of several basiconic sensilla [20], which again corresponds with the olfactory function of the basiconic sensilla on the beetle’s adult antenna [5].

The gross structure of the larval antennae, with three distinguishable segments, is also described in the red flour beetle’s close relative *T. molitor* [21] and other beetles [22–24]. It is similar to the structure of the larval antennae of *M. sexta* [25, 26], whereas flies possess functionally but not serially homolog appendages (dorsal organs) [27, 28]. Interestingly, lepidopterans and dipterans are phylogenetic sister groups [76]. Therefore, the presence of an elaborate larval antenna seems to be the more ancestral form. The current scientific picture sees the adult antennae of holometabolous insects as separate structures that develop from imaginal discs [29–31, 77–80], while the antennae of hemimetabolous insects develop gradually through larval molts until the adult stage [80, 81]. However, this derives from only a few species in essentially two orders: Diptera [29, 30, 79] and Lepidoptera [31, 77, 78, 82–89]. Both belong to the same phylogenetic branch, which is the sister branch to that including the Coleoptera [76].

In general, the development of adult structures from imaginal discs or cells in holometabolous insects is highly derived. In the more ancestral state, it is discussed that cells of the larval appendages are used to build their adult equivalents [34]. In the red flour beetle, this ancestral state is found during the development of the adult legs, which are built from reused polymorphic larval cells, while the legs of *D. melanogaster* are built from imaginal discs and those of *M. sexta* employing a mixture of reused larval cells and imaginal cells [35]. Consequently, it is concluded that the red flour beetle also lacks antennal imaginal discs from which the antenna may arise [36]. Our results indeed support the conclusion that the adult antennae of *T. castaneum* are not formed de novo from imaginal cells/discs but by reuse of polymorphic larval cells. We observed that during the last larval instar, the CSNs retract from the larval antennae and relocate into the head capsule, rather than dissolve as described in *D. melanogaster* [90, 91]. In the prepupal stage, they are then found in the tip of the freshly formed adult antennae (Fig. 4)—by this point still within the larval head cuticle (Additional file 1: Figure S1).

At pupal stage P0%, CSNs (Fig. 2) including OSNs (Fig. 3) are already located in the three distal segments of the flagellum, as they are in the adult beetle [5]. This finding is a major difference to results in *D. melanogaster*, where OSNs are not found in the antennae of fresh pupae but first at about 15% of metamorphosis [90]. The OSN number then obviously rises until pupal stage P50%, when the gross distribution is akin to the adult one. We found that, in all pupal stages, the antennal nerves are present and already or still at P0% axonal terminals of the OSNs are found in the AL and remain detectable in all further pupal stages. This again contrasts results in *D. melanogaster*, where OSNs first reach and enter the AL at about P20% [90, 92], which is also found in *M. sexta* [93, 94], while they reach the AL at about P10% in *A. mellifera* [95].

This persistence of the OSN axons in the ALs, as well as in the GOC and LGs (Additional file 2: Figure S2) leads us to the assumption that those neurons might serve as guidance for the newly born sensory neurons.

### Formation of the olfactory glomeruli

Direct comparison of the f-actin staining via phalloidin and the immunostaining against synapsin showed that during most metamorphic stages, the AL glomeruli were more readily visible in the f-actin staining. For the analysis of the GOCs development, we therefore only used phalloidin staining of f-actin.

In general, f-actin seems to be heavily aggregated in olfactory glomeruli [96]. The same study showed that while f-actin in vertebrates is mostly located on the postsynaptic site, this is not true for insects. Examples from *M. sexta* and *A. mellifera* clearly show that the OSNs are labeled by phalloidin. They further showed that projection neurons do not contribute to the phalloidin staining within the glomeruli. Besides, f-actin serves a key role in neuronal growth and regulation of synaptic vesicle dynamics [97, 98]. Transferring these findings to the beetle, we conclude that the OSNs are in the first place responsible for the formation of glomeruli. This conclusion is supported by results from *M. sexta*, where de-antennation, and therefore lack of OSNs, prevents the formation of the olfactory glomeruli [42]. Further, results from ants clearly show that, without OSNs, the ALs are heavily reduced [43–45].

Unlike *M. sexta* [99] and *A. mellifera* [38], but similar to *D. melanogaster* [100], the larvae of *T. castaneum* possess glomerular organized ALs (Fig. 6A). Like the ALs, also the GOC in larvae is glomerularly organized (Fig. 10A). The larval ALs glomeruli dissolve before pupation, and as in other insects, e.g., *M. sexta* [101–104], *A. mellifera* [95, 105], or *D. melanogaster* [106, 107], the adult AL glomeruli form in the middle of metamorphosis (Fig. 11). Similarly, the adult glomeruli in the GOC form during metamorphosis (Fig. 10).

The glomeruli of the ALs and GOC become first visible in the f-actin staining at 40% of metamorphosis. Considering the functions of f-actin in neuronal growth and regulating synaptic vesicle dynamics [97, 98], at this time, the cytoskeleton of the synaptic structures within the ALs is likely formed. Since the AL glomeruli are first visible in the synapsin staining at P50%, it seems convincing, that only then, synaptic vesicles are recruited, and the first functional synapses are formed.

In *M. sexta,* the process of glomeruli formation was shown to be triggered by a rising 20E titer in the hemolymph [108]. This rise is also present in pupae of *T. castaneum*. In the beetle, a sharp titer increase occurs between P40% and P50% [109]. With roughly 70, the number of glomeruli found at P50% resembles the number found in freshly eclosed beetles [70], increasing to about 90 glomeruli in 7-day-old beetles [5]. Therefore, a basic set of AL glomeruli seems to be genetically encoded and built during metamorphosis. However, after adult eclosion, modifications seemingly occur.

We find Orco expression in the OSNs during all metamorphic stages, which is similar to results in the lepidopteran *Spodoptera litura* (cotton leafworm) [110] and the hymenopteran *Ooceraea biroi* (clonal raider ant) [43]. In contrast, in the dipteran *D. melanogaster,* Orco vanishes before pupation and becomes first detectable again after the formation of the AL glomeruli at about P80% [7] (Fig. 11). This led to the conclusion that Orco, and therefore functional olfactory receptors, are not necessary for the formation of AL glomeruli [7, 46, 47]. In ants, the lack of Orco leads to heavily reduced glomeruli numbers in the AL and total numbers of OSNs [44, 45]. Therefore, the authors conclude that the reduced ALs are an effect of the missing OSNs rather than a direct effect of Orco lacking, which is also supported by a more recent study [43].

To learn about the role of Orco during the metamorphic development in the red flour beetle, we used RNAi interference (RNAi). This results in a nearly complete knockdown of Orco, which results in a massive reduction of Orco-dependent odor responses [10]. Contrasting a knock-out, which is generally present from embryogenesis onwards, the RNAi-mediated knockdown, induced by dsRNA injection [5, 10, 111], has the advantage to be induced at any time. For example, as we did, just before pupation in late larvae. We find that at A0, glomerulization is still clearly visible in knockdown beetles (Fig. 7A), while a heavily reduced glomerulization could be observed at A7 (Fig. 7C). Notably, at both ages, the OSNs still locate normally in the antennae (Fig. 8A; Additional file 6: Figure S4 A). Therefore, we suggest that Orco is not necessary for the initial formation of olfactory glomeruli and their maturation during metamorphosis. However, OR/Orco driven olfactory activity seems to be necessary during the differentiation and adaption of the olfactory system after adult eclosion.

### Origin and metamorphosis of the AL local neurons (LNs)

The vast majority of the AL LNs are GABAergic [13, 37, 60, 69] but also express various neuropeptides, which may also provide an estimate for LN numbers [13, 66, 67, 71, 112, 113].

In the red flour beetle, we identified GABAergic neurons by immunohistochemical staining of GAD. Labeled LNs locate in a cluster lateral to the AL, which is comparable to the “antero-dorsal DC cluster” (CL7) described in *Tenebrio molitor* [39]. In the first half of metamorphosis, the number of labeled neurons is relatively stable. It is first, after glomeruli formation, at mid-metamorphosis, that their number rises. We used neurogenesis detection via the EdU technique [10] to reveal the origin of the rising LN numbers and did not find evidence for newly born neurons. Thus, we conclude that all AL LNs in the red flour beetle are of larval or embryonic origin and gain transmitter identity during metamorphosis. Further, the majority of LNs being recruited coinciding with glomeruli formation might be a common feature, as also most LNs of *M. sexta* ALs seem to be recruited just after the onset of glomeruli formation [112–114].

Similar to GABA immunostaining in *T. molitor* [39], we could first observe a glomerular pattern in the GAD staining in adult *T. castaneum*. Nevertheless, immunoreactive fibers were already visible within the AL volume at stage P30% (Fig. 9A), which corresponds to results from *M. sexta*, where GABAergic fibers are present in the AL at P20% [115]. In *M. sexta*, AST-A immunoreactive fibers occur at stage P10% [112]—just when the LNs start innervating the AL [93]. Similarly, in the honeybee’s ALs, AST-A immunoreactive fibers are found at stage P10% (Additional file 7: Figure S5 A). In *M. sexta* both, GABA and AST-A fibers enter the forming glomeruli at P35% [112, 115]. The same is true for AST-A immunoreactive fibers in the honey bee, which enter the forming glomeruli around P40% (Additional file 7: Figure S5 D). Thus, the LN fibers entering the ALs before glomeruli formation seem to be a common feature.

## Conclusions

In our study, we provide evidence that the adult antennae of the red flour beetle are built from reused polymorphic larval cells, that the CSNs of the beetle’s larval antennae are reused in the adult antennae, and that the larval antennal lobe gets transformed into its adult version. OSN axons are present in the ALs during the whole process. Further, we find that Orco is seemingly not necessary during the initial formation of the AL glomeruli, while the activity of Orco expressing OSNs seems to be required during differentiation after adult eclosion. Comparing our results from the beetle to other model insects, it seems that some features, such as the timepoint of adult glomeruli formation or ingrowth of the AL LNs, are common among insects, while others, e.g., development of sensory appendages or the role of Orco seem to differ. These differences among species should be a reminder to be careful on using generalizations derived from results in a specific insect.

## Methods

### Animals

Experiments were performed using red flour beetles (*Tribolium castaneum*, Herbst 1797; Insecta, Coleoptera) of the wild-type strain “San Bernadino” [116], the transgenic EF1-B-DsRed line (elongation factor1-alpha regulatory region-DsRedExpress; kindly provided by Michalis Averof, Institut de Génomique Fonctionnelle de Lyon, France) [5, 73], or the partial Orco-Gal4 line [5].

The beetles were bred under constant darkness at about 30°C (wildtype) or 28°C (transgenes) and 40–50% relative humidity on organic whole grain wheat flour supplemented with 5% dried yeast powder and 0.05% Fumagilin-B (Medivet Pharmaceuticals Ltd., High River, Alberta, Canada) to prevent sporozoan infections [117].

Pupae of the San Bernadino wildtype were staged using external markers, like eye development and sclerotization of elytra and appendages using a refined version (Additional file 8: Figure S6) of a previously published staging scheme [118, 119]. Due to missing eye color, transgenic beetles were collected at P0% and reared to the desired ages according to a conversion table based upon data collected by time-lapse recordings of total metamorphosis at 28°C.

For the Orco-knockdown experiments, injected individuals were separated as pupae into 5-ml glass vials containing about a 2-g substrate and reared to the desired age.

For the bee experiments, we used the western honeybees (*Apis mellifera*). Honeybee breeding combs (kindly provided by Stefan Berg and Ralph Buechler, Bieneninstitut Kirchhain, Landesbetrieb Landwirtschaft Hessen, Kirchhain, Germany; and Wolfgang Roessler; University of Wuerzburg, Wuerzburg, Germany) were kept under constant darkness at about 34°C, and individual pupae were removed from their comb and staged against a previously published scheme [105].

### EdU injections

5-Ethynyl-2′-desoxuridine (EdU) injections followed a previously published protocol [10]. Cold anesthetized larvae and pupae of different ages were placed in a chilled metal block. Injection of a 100-μM EdU-solution was performed using glass micropipettes made from thin-walled glass capillaries (TW150-4, World Precision Instruments, Sarasota, FL, USA; micropipette puller: Sutter Model P-30, Sutter Instrument, Novato, CA, USA) attached to a pressure ejection system (PDES-02T; npi electronics, Tamm, Germany) until individuals were slightly inflated. After injection, the beetles were transferred into a para-film sealed Petri dish and incubated at 28°C.

### Immunohistochemistry and EdU detection

EdU detections, as well as immunohistochemistry, followed previously published protocols (*T. castaneum* [10, 70]; *A. mellifera* [120]).

For histochemical analysis, dissected ganglia were fixed in either 4% paraformaldehyde or 4% formaldehyde. Due to their larger size, after fixation, honeybee brains were embedded into a gelatin/albumin medium which was hardened overnight in 4 or 8% formaldehyde in PBS at 4°C. Afterward, blocks were cut into 40-μm sections using a vibratome (VT1000S, Leica Biosystems, Nussloch, Germany).

Blocking was performed either in 5% normal goat serum (NGS; Jackson ImmunoResearch, Westgrove, PA, USA) or normal donkey serum (NDS; Jackson ImmunoResearch) based on the primary antisera (for concentrations and details see Table 1).

**Table 1:** Overview of used antibodies and markers

Name	Abbreviation	Host species	Dilution	Vendor/donor (catalogue #, batch #, RRID/CAS #)	References	Specificity
5-Ethynyl-2′-desoxuridine	EdU		100 μM	Thermo Fischer Scientific, Rockford, IL, USA (A10044; 1259424; 61135-33-9)	[122, 123]	
Alexa Fluor 488-coupled phalloidin	Phalloidin		1:200	Thermo Fischer Scientific, Rockford, IL, USA (A12379; n/a; n/a)	[124]	
Alexa Fluor 488 Azide	488-azide		1 μM	Thermo Fischer Scientific, Rockford, IL, USA (A10260; 1320994; n/a)		
Cy2-coupled donkey anti-sheep	DAS-Cy2	Donkey	1:300	Jackson ImmunoResearch; Westgrove, PA, USA (713-225-147, n/a, AB_2340735)		
Cy3-coupled goat anti-chicken	GACh-Cy3	Goat	1:300	Jackson ImmunoResearch; Westgrove, PA, USA (103-165-155, 93117 / 139580, AB_2337386)		
Cy3-coupled goat anti-rabbit	GAR-Cy3	Goat	1:300	Jackson ImmunoResearch; Westgrove, PA, USA (111-165-144, n/a, AB_2338006)		
Cy5-coupled donkey anti-mouse	DAS-Cy5	Donkey	1:300	Jackson ImmunoResearch; Westgrove, PA, USA (715-005-150,132236, RB_2340758)		
Cy5-coupled goat anti-mouse	GAM-Cy5	Goat	1:300	Jackson ImmunoResearch; Westgrove, PA, USA (115-175-146, n/a, AB_2338713)		
Cy5-Sulfo Azide	Cy5-azide		1 μM	Jena Bioscience, Jena, Germany (CLK-AZ118-1; Kli009-030; n/a)		
*Diploptera punctata* Allatostatin I	Dip-AST	Rabbit	1:20,000	H.J. Agricola (Friedrich Schiller University, Jena, Germany) (n/a, n/a, AB_2314318)	[125]	Ame: [71]
*Drosophila melanogaster* Synapsin I (SYNORF1)	Synapsin	Mouse	1:50	E. Bucher, University of Würzburg, Germany (n/a, n/a, AB_2313617)	[126]	Ame: [127] Tcas: [113]
HRP-coupled donkey anti-sheep	DAS-HRP	Donkey	1:1,000	Jackson ImmunoResearch; Westgrove, PA, USA (713-035-147, 69205, AB_2340710)		
HRP-coupled goat anti-rabbit	GAR-HRP	Goat	1:1,000	Jackson ImmunoResearch; Westgrove, PA, USA (111-035-003, 130223, AB_2313567)		
Moth-R2, Orco antiserum	Moth-R2	Rabbit	1:5,000	J. Krieger, University Halle-Wittenberg, Germany (n/a; n/a; n/a)	[5]	Tcas: [5]
*Rattus norvegicus* glutamate decarboxylase (rabbit)	GADr	Rabbit	1:1,000	Sigma-Aldrich; now Merck KGaA, Darmstadt, Germany (G5163; 113M4772; AB_477019)		Tcas: This study by Western blot
*Rattus norvegicus* glutamate decarboxylase (sheep)	GADs	Sheep	1:5,000	W. Oertel, Laboratory of Clinical Science, Mansfield, MA, USA (n/a; n/a; AB_2314497)	[128]	Tcas: This study by Western blot
Red fluorescent protein	RFP	Chicken	1:3,000	Rockland Immunochemicals INC, Limerick, PA, USA (600-901-379, 26274, AB_10704808)		

Wholemounts of ganglia were mounted either aqueous in the Mowiol [121] or after dehydration in an ascending ethanol series and clearing with methyl salicylate (Merck KGaA, Darmstadt, Germany) in Permount mounting medium (Fisher Scientific, Pittsburgh, PA) between two coverslips using reinforcing rings as spacers to prevent squeezing. Vibratome sections were dehydrated in an ascending ethanol series and cleared in xylol, before being mounted in Entellan (Merck) between a microscope slide and a coverslip.

### Western blotting

To demonstrate the specificity of the used anti-GAD antibodies in *T. castaneum*, western blot analysis was performed as previously described [113]. Twenty brains were dissected and homogenized in 20 μl reducing sample buffer and boiled for 5 min. A 10 μl of the sample was loaded and run on a discontinuous 10% SDS polyacrylamide gel and blotted onto Optitran BA-S 83 nitrocellulose membranes (Carl Roth GmbH & Co. KG, Karlsruhe, Germany). After blocking, the membrane was incubated with the GAD antisera (1:10,000) overnight at 4°C, washed, and incubated with HRP conjugated anti-sheep/rabbit secondary antibody (1:1,000; see Table 1) for 2 h at room temperature. Finally, the blot was incubated with chemiluminescent substrate (SuperSignal™ West Pico, Thermo Fischer Scientific, Rockford, IL, USA) and either exposed to Amersham Hyperfilm ECL (GE Healthcare Europe GmbH, Freiburg, Germany) and digitized with a flatbed scanner (9900F Mark II, Canon Inc, Tokyo, Japan) or imaged using a CCD image system (Image Station 440CF, Kodak Digital Science, Rochester, NY, USA). A single band at about 55 kDa was recognized for the sheep antibody, as well as for the rabbit antibody matching to the predicted size of Tcas-GAD (UniProt ID: D6WRJ1) of about 58 kDa (Additional file 9: Figure S7).

### Orco-knockdown

*Tcas-orco-5′* (1067 bp) dsRNA (*RNAi*^*Orco*^) and *Cmor-MIP2* dsRNA (*RNAi*^*Sham*^) were synthesized from PCR templates following a previously published protocol [5], using the HiScribe T7 High Yield RNA Synthesis Kit (New England Biolabs, Ipswich, MA, ISA). Both dsRNAs (about 0.3 to 0.5 μg/μl in injection buffer) were injected with the same setup as used for EdU injection into last-stage larvae (LL) until individuals were slightly stretched. The Orco knockdown was verified by immunohistochemistry against Orco (Moth-R2, kindly provided by J. Krieger, University of Hohenheim, Germany) on cryo-sections of antennae (Additional file 6: Figure S4) as previously published [5, 10].

### Image acquisition and analysis

Fluorescent preparations were imaged using a confocal laser scanning microscope (TCS SP2 or TCS SP5, Leica Microsystems, Wetzlar, Germany) and analyzed with Amira 6.5 graphics software (FEI SAS a part of Thermo Fisher Scientific, Mérignac Cedex, France). In Amira, AL glomeruli numbers were acquired through manual 3D reconstruction and LN cell bodies were manually counted using the “landmark” tool.

Images of larvae and pupae were acquired in Progress Capture Pro 2.10 (Jenoptik, Jena, Germany) using a CCD camera (Progress C4 or C12plus, Jenoptik) attached to a (fluorescence) stereomicroscope (Stereo Lumar.V12, Carl Zeiss Microscopy, Jena Germany; Wild M3, Herbrugg, CH).

Further image processing (global level adjustments, contrast, and brightness optimization) was performed in Photoshop CC (Adobe Systems, San Jose, CA, USA), while final figure arrangements were made in Illustrator CC (Adobe Systems).

For basic statistics (arithmetic mean and standard deviation) on the number of immunoreactive local neurons, we used Excel 2019 (Microsoft Corporation, Redmond, WA, USA).

### Time-lapse series

Time-lapse series images were acquired as stated above for larvae and pupae, but in a temperature-controlled environment at about 30°C. Afterward, images were further processed (cropping, global level adjustments; contrast, and brightness optimization) in Photoshop CC. Graphical annotations were prepared in Illustrator CC and final video assembly, and annotations were performed using Premiere CC (Adobe Systems).

## Supplementary Information

**Additional file 1: Figure S1.** Localization of the adult appendages and sensory neurons in the head capsule of the prepupa. (A-A') Stereo microscopic image in ventral view of a prepupa with the opened larval head capsule, showing the location of the adult appendages within the prepupal head capsule, as well as the location of the CSNs in the adult antennae. (B) Schematic drawing of the location of the adult head within the prepupal head capsule in dorsal view. (C, D) Confocal image of the DsRed reporter signal (magenta) of the EF-1-B-line (C) and Orco-Gal4xUAS-DsRed-line (D), showing the position of the CSNs / OSNs cell cluster in the intact head capsule of prepupae. Scale bars 50 μm.

**Additional file 2: Figure S2.** OSNs in primary processing centers at P0%. Representative optical slices showing the DsRed reporter signal (magenta) of the Orco-GAL4 line, indicating OSNs, counterstained with phalloidin (green) to visualize the general neuroarchitecture. Scale bars 10 μm.

**Additional file 5: Figure S3.** Orco in the antennae before glomeruli formation. Confocal maximum projection of 50μm slice a P10% antenna showing OSNs labeled by immunohistochemistry using the crossreactive Moth-R2 antiserum. Scale bars 50μm.

**Additional file 6: Figure S4.** Immunohistochemical Orco knock-down verification. Representative maximum projections of 50 μm cry-sections of the antennae of freshly eclosed (A0) beetles of the CSN-labeling EF-1-B-DsRed line after (A) *RNAi*^*Orco*^ and (B) *RNAi*^*Sham*^ injection. (A – A’’, B – B’’) The DsRed reporter signal is depicted in green, while Orco immunostaining is depicted in magenta. This channel also includes the autofluorescence of the antennal cuticle. In both treatment groups, the gross CSN distribution is very similar, while Orco cannot be detected in the *RNAi*^*Orco*^ group (A). Scale bars 20 μm.

**Additional file 7: Figure S5.** Development of AST-A immunoreactivity in the AL of *Apis mellifera*. Representative optical slices of AST-A immunoreactivity in the AL of A. mellifera workers at different developmental stages. (A) In the AL of P10% pupae, AST-A fibers are restricted to the lateral portion of the AL. (B, C) At P20% AST-A fibers penetrate the AL. (D) At P40% immunoreactive fibers locate in most of the forming glomeruli. (E-H) From P50% AST-A immunoreactivity shows clearly distinguishable glomeruli, which grow until adult eclosion. Scale bars 40μm.

**Additional file 8: Figure S6.** Staging of wild-type beetles during metamorphosis. The comparison of time-lapse recordings of nine pupae led to an averaged time for the metamorphosis of 126 h (5,25 d) at 30°C with a deviation of 5,3 h. The development of the eyes [118, 119], as well as the sclerotization of mandibles, elytra, and legs, served as external markers, in a time-dependent context. The fresh eclosed pupae are brighter and glossy with a maximum of three rows of ommatidia. After about 20% (25 h after pupa formation (APF)), about six rows of ommatidia are visible and form a kidney-shaped eye. At 30% (40 h APF) the formation of the seventh row is in progress and the distance between the ommatidia shrinks. At about 50% all ommatidia are visible and outgrowth to the sides of the antennal pocket, thus the eyes look horseshoe-shaped. After 68% (86 h APF; SD 2,6 h) the outlines of ommatidia are resolved and the eye appears homogeneous. Besides the eye, at 76% (96 h APF, SD 3,6 h) the majority of mandibles are amber followed by the coloration of the elytra at 85% (106 h APF, SD 2,9 h) and sclerotization of the legs and antennae at 91% (114 h APF, SD 3.3). Finally, the imago eclosed after 126 h (SD 5.3 h).

**Additional file 9: Figure S7.** Specificity of the used antisera against GAD. Western blot analysis on *Tribolium castaneum* brain tissue shows a single band of about 55 kDa for both antibodies which corresponds to the predicted size of Tcas-GAD (UniProt ID: D6WRJ1) of about 58 kDa.

## Data Availability

The datasets generated and/or analyzed during the current study are either included in this published article and its additional files and/or available in the University of Marburg’s institutional data (data_UMR) repository at https://data.uni-marburg.de/handle/dataumr/73 [129–137].
