# Supplementary figures and images for: Metamorphic development of the olfactory system in the red flour beetle (Tribolium castaneum, Herbst)
Source: BMC Biol. 2021 Jul 30;19:155. doi: 10.1186/s12915-021-01055-8 (PMC8323255; doi:10.1186/s12915-021-01055-8)

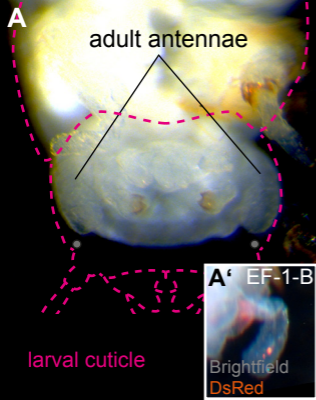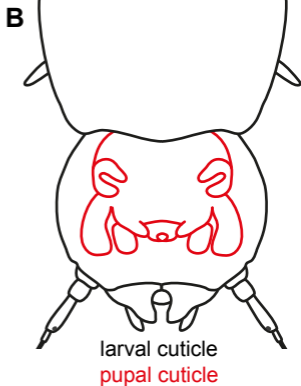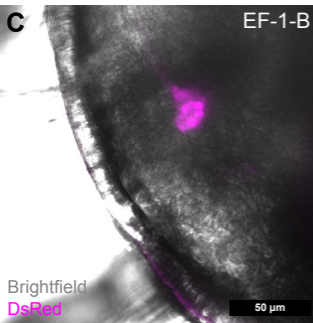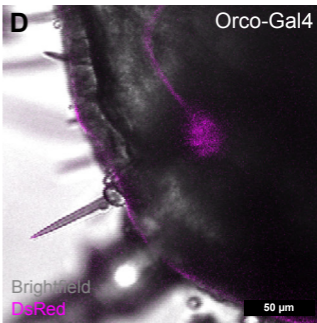

Supplement: Supplementary file 3 — Additional file 1: Figure S1. Localization of the adult appendages and sensory neurons in the head capsule of the prepupa. (A-A') Stereo microscopic image in ventral view of a prepupa with the opened larval head capsule, showing the location of the adult appendages within the prepupal head capsule, as well as the location of the CSNs in the adult antennae. (B) Schematic drawing of the location of the adult head within the prepupal head capsule in dorsal view. (C, D) Confocal image of the DsRed reporter signal (magenta) of the EF-1-B-line (C) and Orco-Gal4xUAS-DsRed-line (D), showing the position of the CSNs / OSNs cell cluster in the intact head capsule of prepupae. Scale bars 50 μm. [file 12915_2021_1055_MOESM1_ESM.pdf]

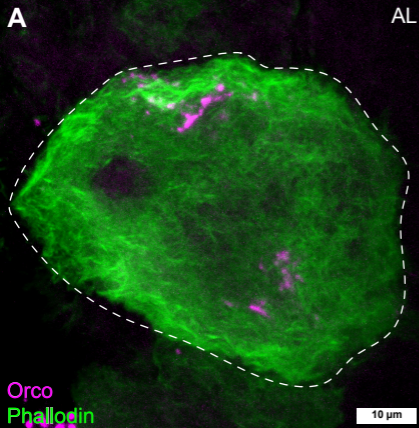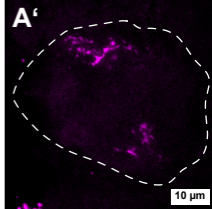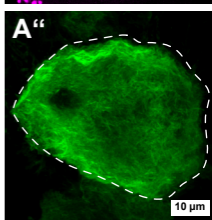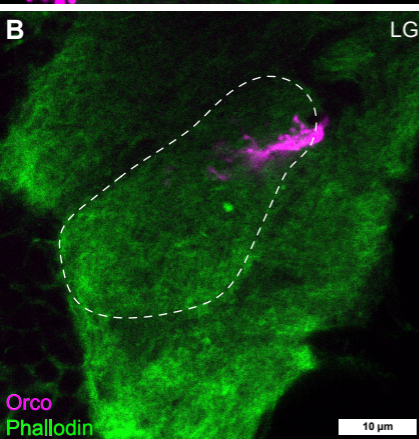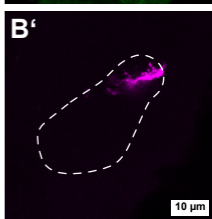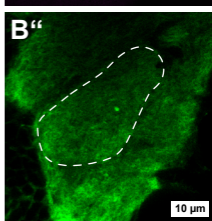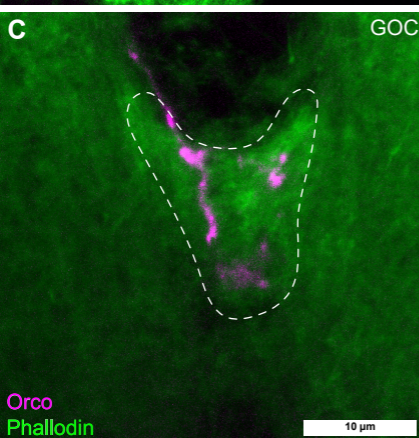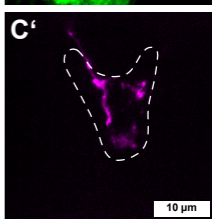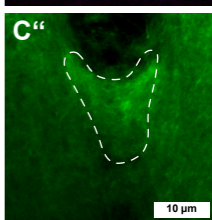

Supplement: Supplementary file 4 — Additional file 2: Figure S2. OSNs in primary processing centers at P0%. Representative optical slices showing the DsRed reporter signal (magenta) of the Orco-GAL4 line, indicating OSNs, counterstained with phalloidin (green) to visualize the general neuroarchitecture. Scale bars 10 μm. [file 12915_2021_1055_MOESM2_ESM.pdf]

**A****P10%****MothR2**  
**DAPI**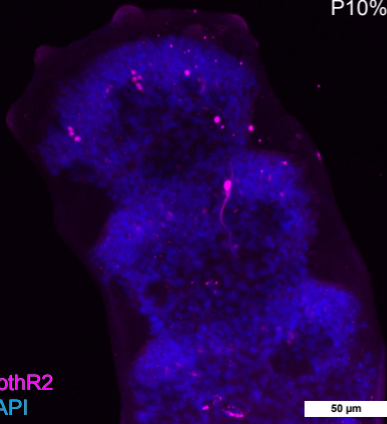**A'**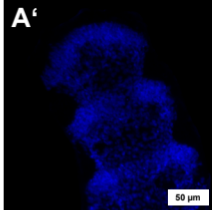**A''**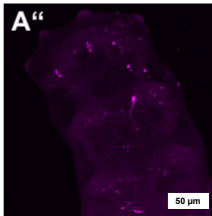

Supplement: Supplementary file 5 — Additional file 5: Figure S3. Orco in the antennae before glomeruli formation. Confocal maximum projection of 50μm slice a P10% antenna showing OSNs labeled by immunohistochemistry using the crossreactive Moth-R2 antiserum. Scale bars 50μm. [file 12915_2021_1055_MOESM5_ESM.pdf]

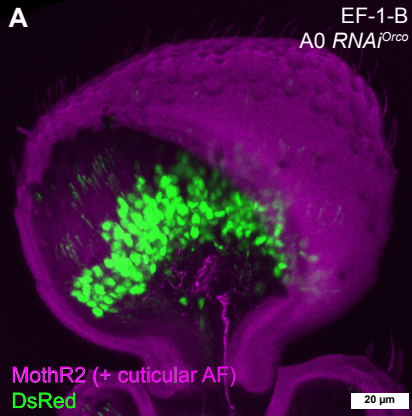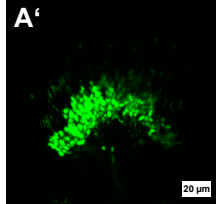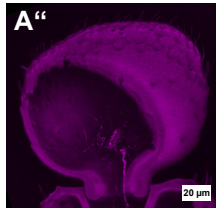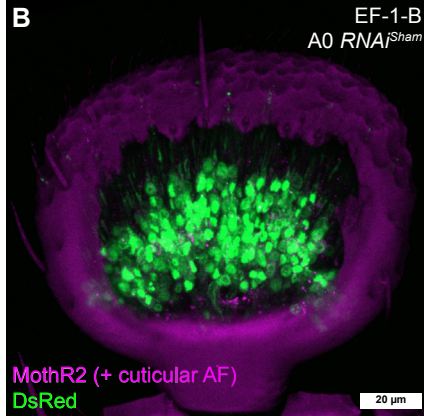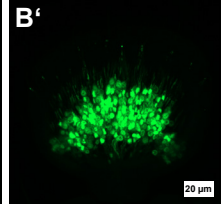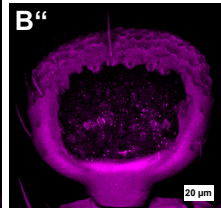

Supplement: Supplementary file 6 — Additional file 6: Figure S4. Immunohistochemical Orco knock-down verification. Representative maximum projections of 50 μm cry-sections of the antennae of freshly eclosed (A0) beetles of the CSN-labeling EF-1-B-DsRed line after (A) RNAiOrco and (B) RNAiSham injection. (A – A’’, B – B’’) The DsRed reporter signal is depicted in green, while Orco immunostaining is depicted in magenta. This channel also includes the autofluorescence of the antennal cuticle. In both treatment groups, the gross CSN distribution is very similar, while Orco cannot be detected in the RNAiOrco group (A). Scale bars 20 μm. [file 12915_2021_1055_MOESM6_ESM.pdf]

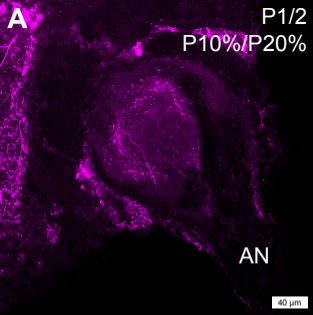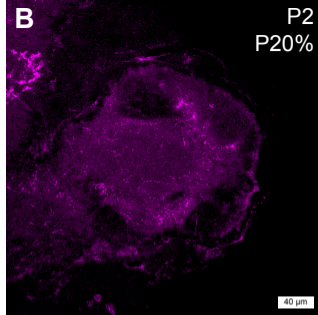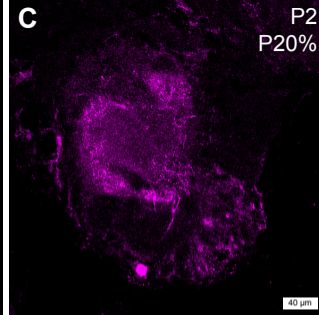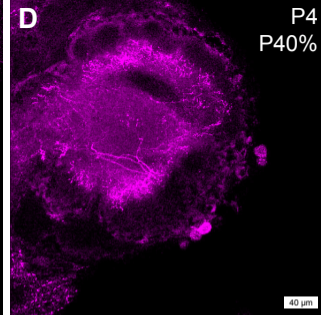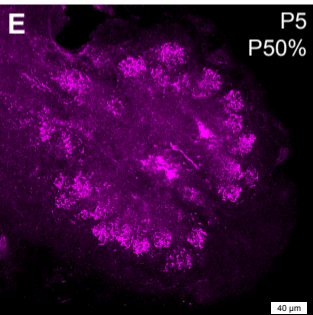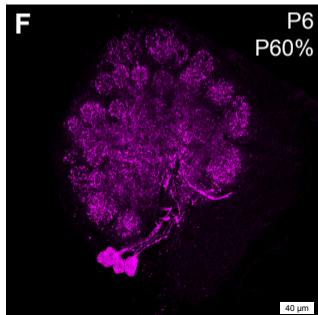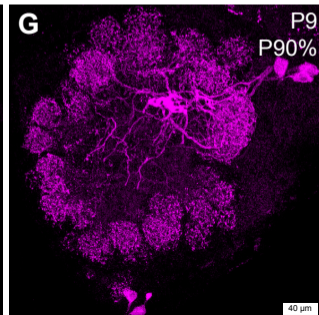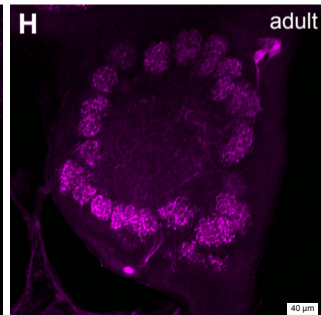

Supplement: Supplementary file 7 — Additional file 7: Figure S5. Development of AST-A immunoreactivity in the AL of Apis mellifera. Representative optical slices of AST-A immunoreactivity in the AL of A. mellifera workers at different developmental stages. (A) In the AL of P10% pupae, AST-A fibers are restricted to the lateral portion of the AL. (B, C) At P20% AST-A fibers penetrate the AL. (D) At P40% immunoreactive fibers locate in most of the forming glomeruli. (E-H) From P50% AST-A immunoreactivity shows clearly distinguishable glomeruli, which grow until adult eclosion. Scale bars 40μm. [file 12915_2021_1055_MOESM7_ESM.pdf]

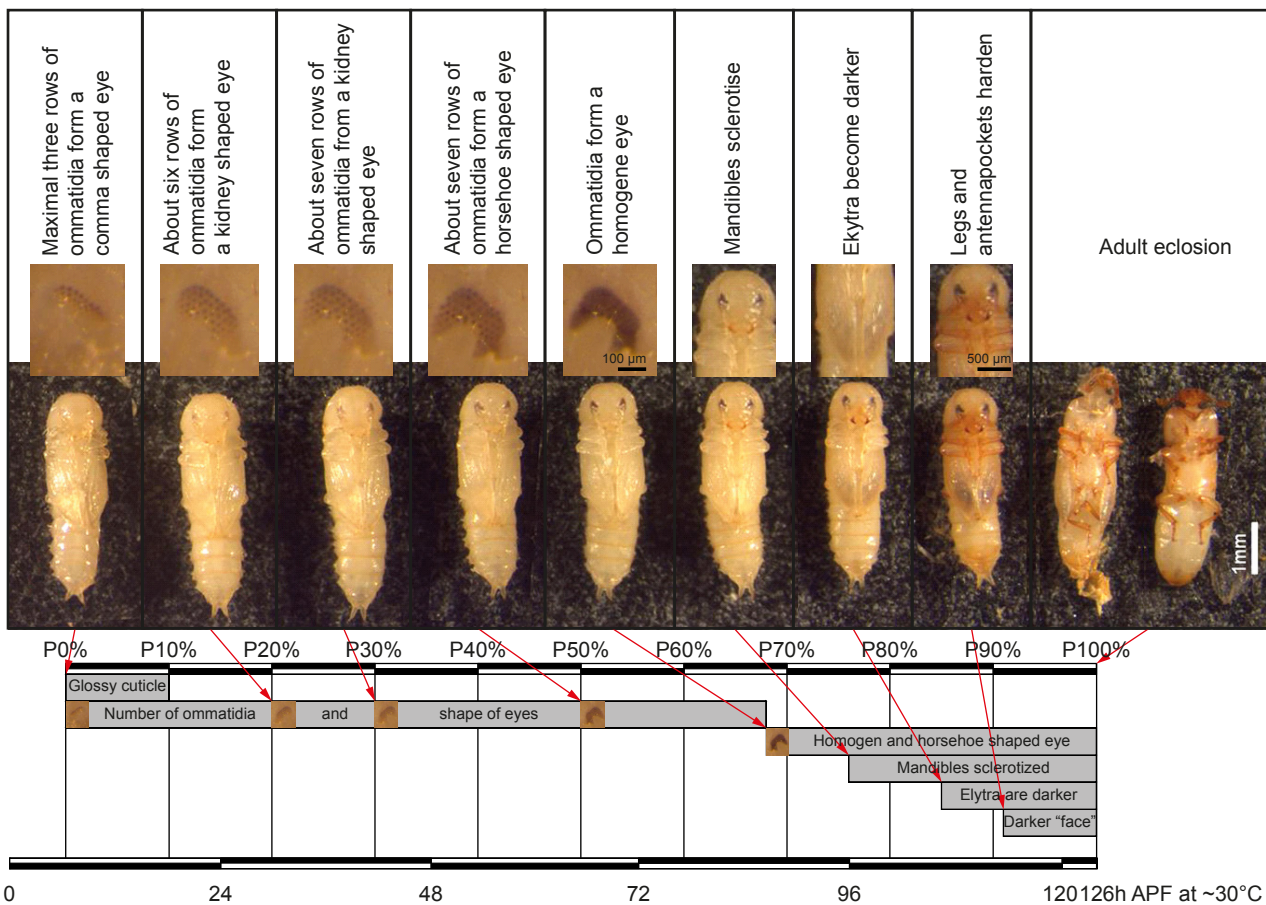

Supplement: Supplementary file 8 — Additional file 8: Figure S6. Staging of wild-type beetles during metamorphosis. The comparison of time-lapse recordings of nine pupae led to an averaged time for the metamorphosis of 126 h (5,25 d) at 30°C with a deviation of 5,3 h. The development of the eyes [118, 119], as well as the sclerotization of mandibles, elytra, and legs, served as external markers, in a time-dependent context. The fresh eclosed pupae are brighter and glossy with a maximum of three rows of ommatidia. After about 20% (25 h after pupa formation (APF)), about six rows of ommatidia are visible and form a kidney-shaped eye. At 30% (40 h APF) the formation of the seventh row is in progress and the distance between the ommatidia shrinks. At about 50% all ommatidia are visible and outgrowth to the sides of the antennal pocket, thus the eyes look horseshoe-shaped. After 68% (86 h APF; SD 2,6 h) the outlines of ommatidia are resolved and the eye appears homogeneous. Besides the eye, at 76% (96 h APF, SD 3,6 h) the majority of mandibles are amber followed by the coloration of the elytra at 85% (106 h APF, SD 2,9 h) and sclerotization of the legs and antennae at 91% (114 h APF, SD 3.3). Finally, the imago eclosed after 126 h (SD 5.3 h). [file 12915_2021_1055_MOESM8_ESM.pdf]

## GADs (Oertel)

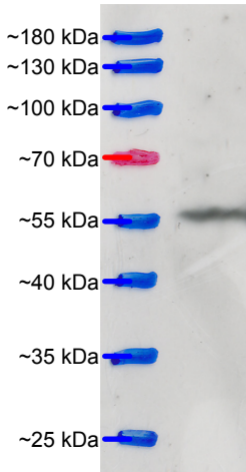

## GADr (Sigma)

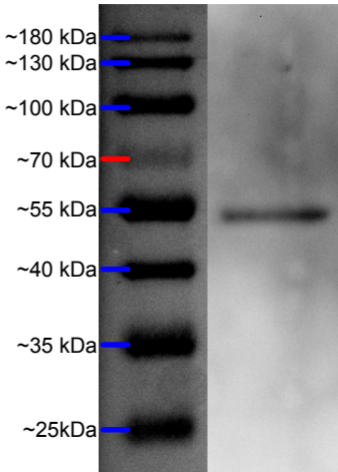

Supplement: Supplementary file 9 — Additional file 9: Figure S7. Specificity of the used antisera against GAD. Western blot analysis on Tribolium castaneum brain tissue shows a single band of about 55 kDa for both antibodies which corresponds to the predicted size of Tcas-GAD (UniProt ID: D6WRJ1) of about 58 kDa. [file 12915_2021_1055_MOESM9_ESM.pdf]
